# Narrowing the gap for city building height predictions

**DOI:** 10.1038/s41598-025-15929-2

**Published:** 2025-08-15

**Authors:** C. Scott Watson, John R. Elliott

**Affiliations:** 1 School of Geography and water@leeds, University of Leeds, Leeds , LS2 9JT UK; 2 School of Earth and Environment, COMET, University of Leeds, Leeds , LS2 9JT UK

**Keywords:** Environmental impact, Sustainability

## Abstract

Understanding the 3D evolution of urban environments at high resolution through space and time is crucial for targeting sustainable development and enhancing resilience to hazards but usually requires expensive commercial satellite or aerial imagery. This leads to data scarcity and analytical biases in countries without access to these capabilities. Here we use high (1.5 m) resolution digital elevation models (DEMs) derived from satellite imagery to measure the vertical component of three cities in the Global South (Nairobi, Kathmandu and Quito), which we evaluate against published datasets of modelled heights. Building heights could be determined to < 1 m mean absolute error (MAE) using the DEMs, and 2.2–7.0 m MAE using a deep learning model trained to predict heights using high-resolution satellite imagery. Google’s Open Buildings 2.5D Temporal Dataset further improved on our deep learning models for two of the three cities, although tended to overestimate building heights. Constraining the building-scale vertical dimension of urban growth creates new opportunities to quantify population distributions, assess natural hazard exposure and vulnerabilities, and evaluate material consumption for sustainable development. Deep learning derived building heights begin to address global inequalities in data availability but should be evaluated locally alongside reference data to determine biases.

## Introduction

The global trend of urbanisation creates cities that are expanding horizontally and vertically, with building stocks that are redeveloping through time^1–3^. The 3D form of urban areas is intrinsically linked to factors including population distribution^4,5^natural and anthropogenic hazards^6^disaster risk management^7,8^building materials consumption^9^and socio-economic processes and governance^10,11^. Vertical expansion can conserve land, mitigating the consumption of greenspaces, as well as optimising infrastructure compared to sprawled cities. However, formal and informal development can increase population exposure to natural hazards by densifying built-up areas. The ability to resolve building-level detail across a city is essential to capture the associated impacts on flood routing^12,13^. Similarly, the vertical component of cities creates microclimates that change as a function of building height due to the interaction between solar radiation shielding and airflow turbulence^14^which also affects pollution dispersion^15^. Building heights also reflect population distributions and informal development, which often occurs in more hazardous areas such as on steep slopes or adjacent to river channels, which means these communities are disproportionately affected by natural hazards^16–18^. Population data underpin the analytics and monitoring for international frameworks including the sustainable development goals (SDGs) and the Sendai Framework for Disaster Risk Reduction^4,19^. However, the lack of globally consistent and high-resolution population data remains a barrier for integration with increasingly detailed hazard models^20,21^. Spatially and temporally consistent building-scale mapping is crucial for advancing both top-down census disaggregation approaches and bottom-up methods of estimating population distribution^22–24^.

The horizontal expansion of urban areas is well studied, but the vertical elongation of cities is a growing facet of the built environment that is less well quantified globally. Satellite-based mapping of horizontal urban growth mapping has been commonplace for several decades, with global products revealing the horizontal sprawl of cities^25–27^ and population distributions^19,28,29^. However, built-up area classifications do not account for the spatial distribution, density, and volume of buildings. Whilst building footprints are often mapped by national organisations, such datasets are often lacking or dated in low- and middle-income countries. Open access datasets such as OpenStreetMap (OSM) are a valuable source of building footprints; however, the completeness is similarly biased towards high-income countries^30^. Recent advances have produced regional-scale building footprint and height datasets derived using deep learning approaches applied to high and medium resolution satellite imagery that can mitigate spatial and temporal biases^31–33^. These models offer to reduce the requirement for access to expensive commercial satellite data, aerial imagery, or LIDAR surveys, which are typically required to build 3D city models. However, deep learning training datasets are similarly spatially biased to areas of data availability, for example North America and Europe^32,34^which highlights the importance of local validation in cities with diverse building forms and structural materials.

To advance efforts in accurately quantifying the vertical dimensions of cities, we aim to comprehensively evaluate existing data products of building footprints and height, alongside our own contribution of height observations and model predictions. We perform city-scale assessments of building footprints and heights by integrating high-resolution satellite-derived digital elevation models (DEMs) with altimetry data. This integrated approach enables us to evaluate the accuracy of building height estimations at the city level using both observational (DEM-derived) and modelling (deep learning) techniques. This differs from other studies, where the absence of building height inventories means reference heights for accuracy assessments are estimated using the number of stories multiplied by a fixed floor height, for example 3 m^35,36^which does not reflect the complexity of residential, commercial, and industrial building types. To achieve our aim, we: (1) assess the completeness and quality of open access building footprints; (2) create unique 3D city models for Nairobi, Quito, and Kathmandu using high-resolution satellite imagery; and (3) train and test the applicability of a deep learning workflow to estimate building, benchmarked against published datasets. Our study cities (Supplementary Fig. 1, 2) formed part of the *Tomorrow’s Cities* project, which aimed to reduce and address inequalities in future urban disaster risk^37^. They vary in land cover, topographic relief, population density, architectural form, and the prevalence of informal settlements. They also reflect contexts where 3D city models are critical for effective urban planning and informing disaster risk reduction strategies, yet where limited historical access to high-resolution imagery poses challenges for data quality and completeness.

## Results

### Building footprint datasets

**Fig. 1:**
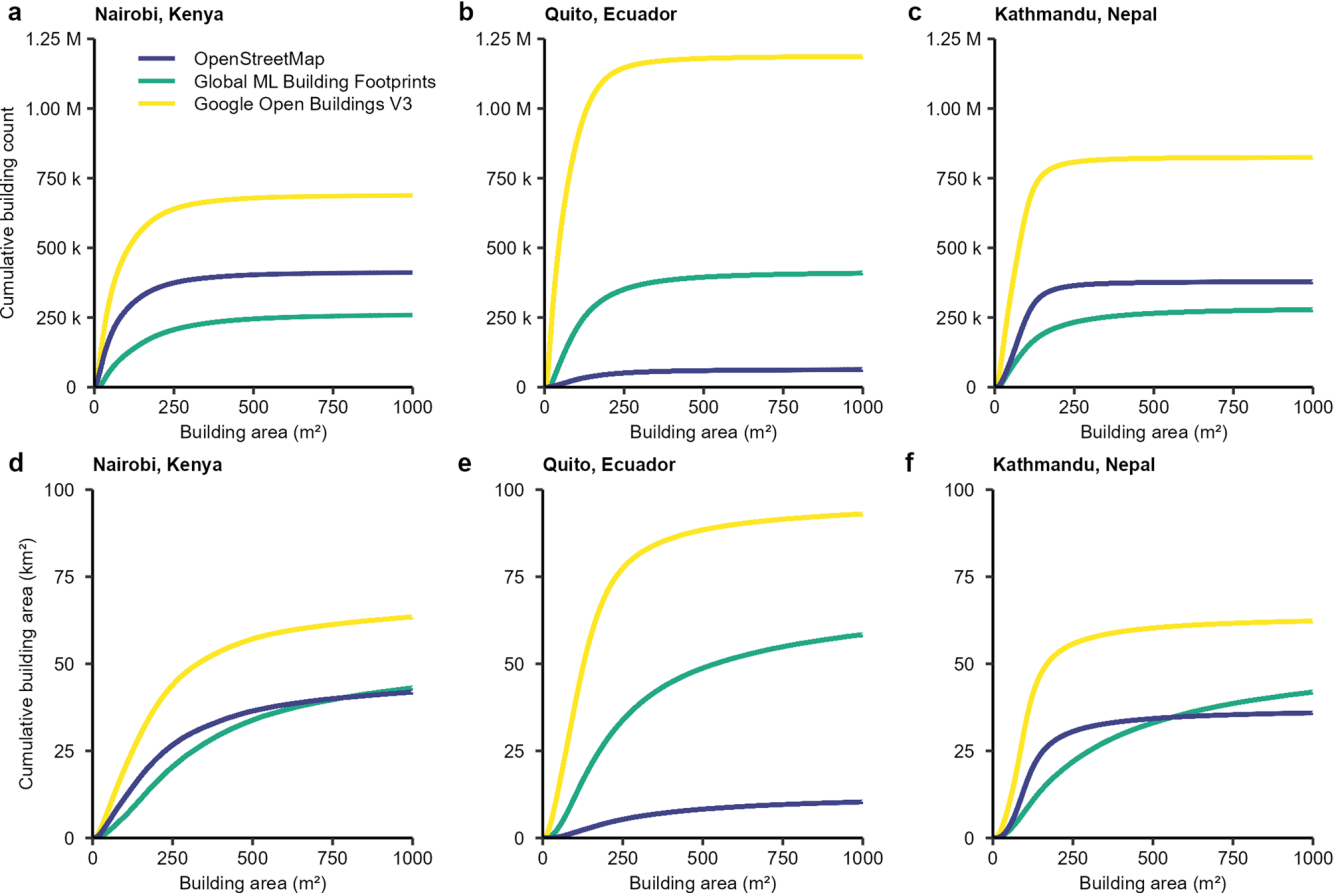
Cumulative counts (**a**–**c**) and areas (**d**–**f**) for building footprint datasets covering each city. Buildings with an area greater than 1,000 m^2^ are not shown but are included in the summary statistics (Table 1).

We observed high variability in the total count and area of building footprints in open access datasets (Fig. 1; Table 1). Google Open Buildings v3 (GOB) had the largest count and areal coverage of buildings across the three study cities. OSM and the Microsoft’s Bing Maps Global ML Building Footprints (GMLBF) were more closely aligned for Nairobi and Kathmandu but not for Quito, where the difference across all three datasets was greatest. In Quito, the building count was over 1.1 million in the GOB dataset compared to 64,309 in OSM, with a large difference in total building area of 101 km^2^ and 15 km^2^ respectively (Table 1). The differences in total building area between the three building footprint datasets were 36%, 148% and 52% for Nairobi, Quito, Kathmandu respectively. The size of buildings mapped in each dataset also varied, with GOB having the smallest median building size for all cities (55–62 m^2^, and GMLBF having the greatest (100–530 m^2^ (Table 1). Although we did not perform a building-scale comparison of each dataset due to their unknown timestamps, comparison with the World Settlement Footprint 2019 data shows the spatially variable completeness of each dataset (Supplementary Fig. 3). This also highlights potential spatial biases, such as the omission of the Mukuru informal settlement in the GMLBF, detection in GOB, and partial mapping in OSM. The settlement is a large area of closely spaced and adjoining small buildings.

**Table 1:** Comparison of open-access Building datasets for each city.

	OpenStreetMap (OSM)			Bing (GMLBF)			Google Open Buildings (GOB)		
	Number of buildings	Total area (km^2^)	Median building size (m^2^)	Number of buildings	Total area (km^2^)	Median building size (m^2^)	Number of buildings	Total area (km^2^)	Median building size (m^2^)
Nairobi	415,179	50.70	60	265,313	58.32	117	692,352	73.17	55
Quito	64,309	15.20	119	417,057	77.67	530	1,189,978	100.97	55
Kathmandu	378,838	37.15	79	283,761	51.76	100	825,251	63.58	62

## Building height observations and modelling

**Fig. 2:**
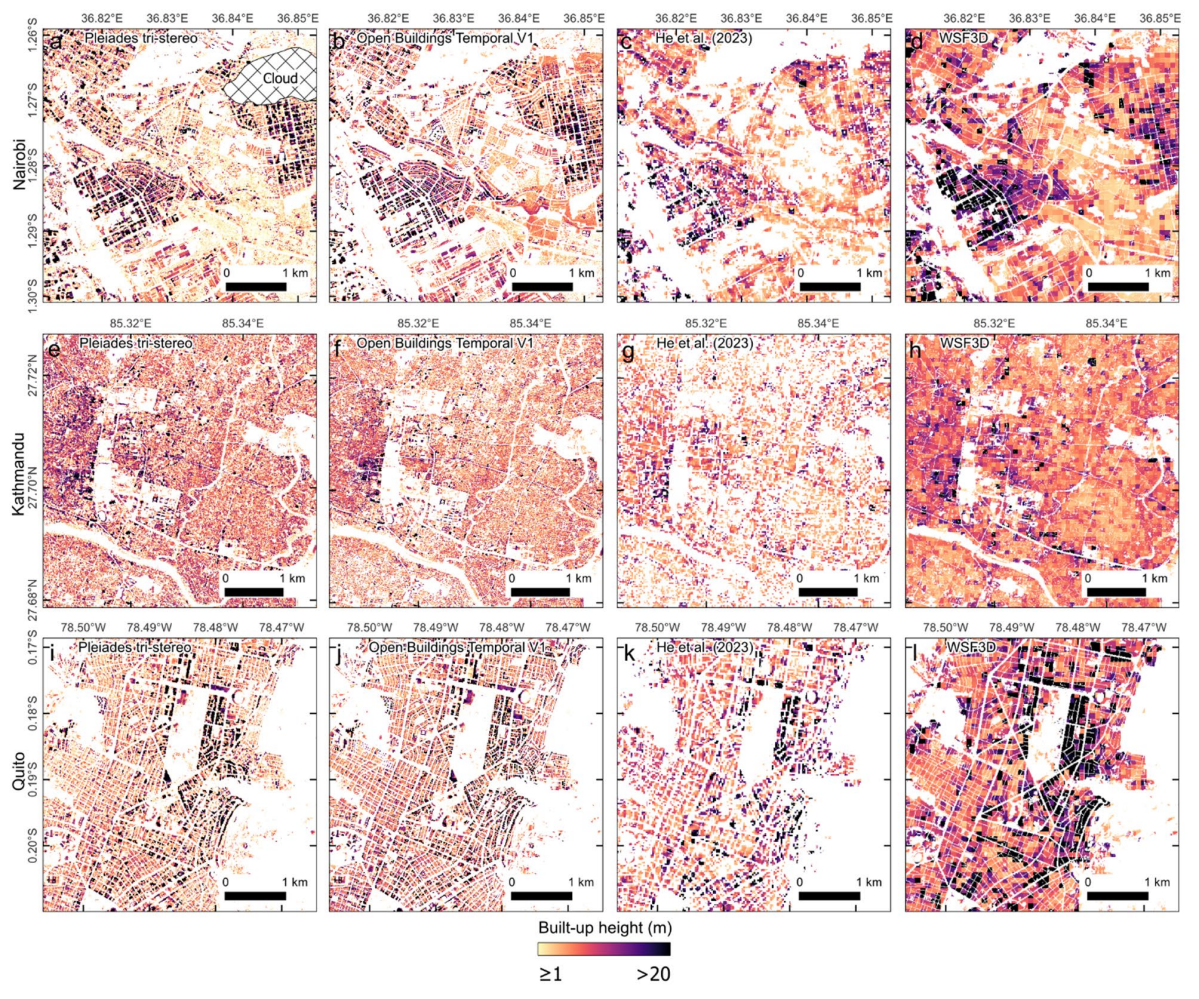
Example built-up area heights shown for areas of Nairobi (first row), Kathmandu (second row), and Quito (third row). The Pleiades-derived heights in this study (**a**, **e**, **i**) are shown alongside other open access datasets for visual comparison (**b**–**d**), (**f**−**h**), (**j**–**l**). Figure created in QGIS 3.28.10^39^.

**Fig. 3:**
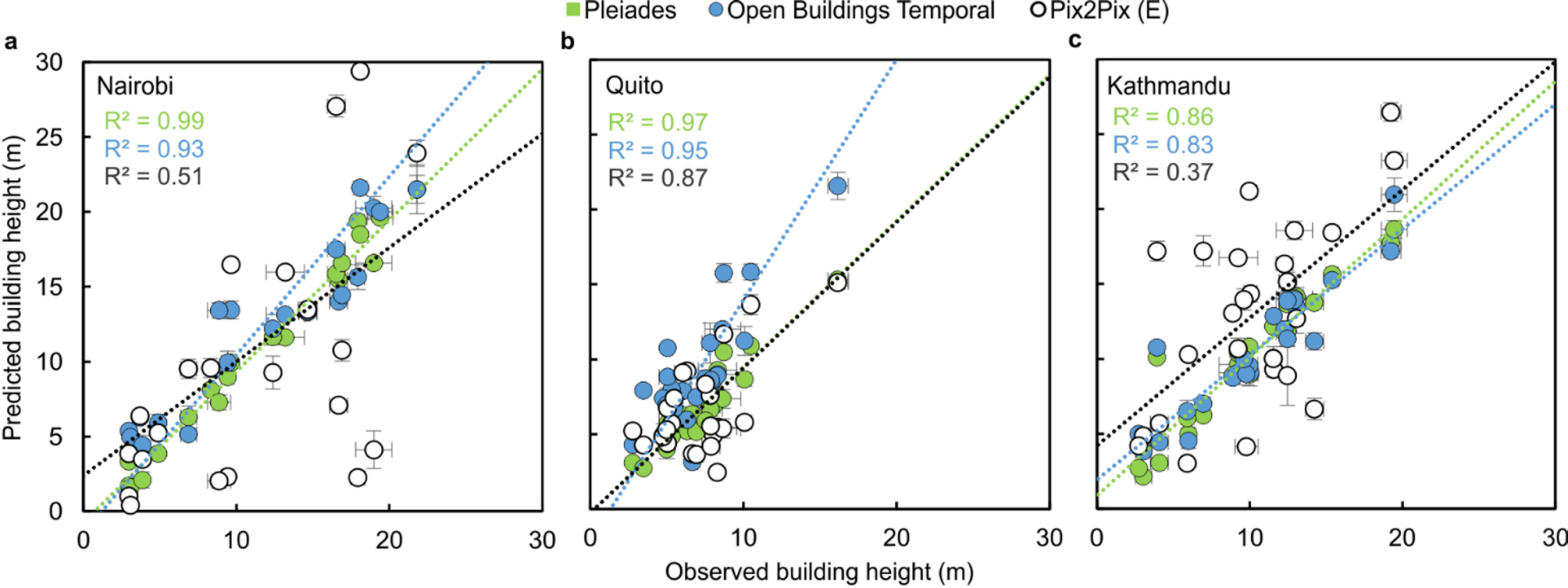
ICESat-2 derived (observed) and predicted building heights for Nairobi (**a**), Quito (**b**), and Kathmandu (**c**). *n* = 25 for each city. Buildings > 30 m tall (*n* = 1 for Quito and *n* = 3 for Nairobi) are not shown but are included in the linear regression. Pleiades (green), Open Buildings Temporal (blue), and Pix2Pix Model E (black) predictions are shown.

The dominant spatial patterns in building height variation were similar between the 3D datasets (Fig. 2). However, the building heights of He et al.^38^ featured more data gaps, particularly over Kathmandu (Fig. 2g). These gaps, which were represented by a height value of 0 m in the dataset, precluded a comparison of this dataset with the satellite laser altimeter ICESat-2 reference heights. The reference heights were derived for 25 buildings in each city and had a mean uncertainty of 0.5 m ± 0.3 m. The Pleiades-derived building heights from stereo optical satellite imagery were closest to the ICESat-2 reference heights across all three cities, with a mean absolute error (MAE) of < 1 m in all cases (Fig. 3; Table 2). The Open Buildings Temporal (OBT) dataset achieved the second-highest accuracy for Nairobi and Kathmandu with a MAE of 2.5 m and 1.2 m respectively, followed by the deep learning Pix2Pix Model E derived in this study (Table 2). Pix2Pix Model E was trained on six high-resolution images of both Nairobi and Kathmandu (Supplementary Table 1) and featured a MAE ranging from 2.2 to 7.0 m. Outliers were particularly evident for this model in Kathmandu and Nairobi (Fig. 3) (R^2^ = 0.37 and 0.51). A linear regression between ICESat-2 and the OBT dataset was more constrained (R^2^ = 0.83–0.95), although OBT displayed a bias to overpredict building heights in Nairobi and Quito, which was not evident in Kathmandu (Fig. 3a). Evaluation of these buildings in Quito did not reveal any apparent reason for the overestimation and the buildings were generally residential building blocks with flat roofs (Supplementary Table 2). The largest difference was for a high-rise residential block, where OBT overpredicted the height by 24 m, yet the Pleiades-derived height was within 1 m of the reference. The magnitude of the differences suggests the discrepancy was not due to redevelopment/ construction of buildings between the reference data and OBT data acquisition, and is potentially related to the imagery used by OBT for the inferencing, which are not known.

**Table 2:** Comparison of observed (derived from ICESat-2) and predicted Building heights. *n* = 25 for each city.

City	Mean absolute error (m)						
	Pleiades	Open buildings temporal	Pix2Pix model				
			A	B	C	D	E
Nairobi	0.92	2.49	10.26	7.72	9.79	9.34	7.02
Kathmandu	0.96	1.21	5.62	4.64	3.78	3.64	4.63
Quito	0.94	3.34	4.50	4.81	3.58	4.68	2.16

**Fig. 4:**
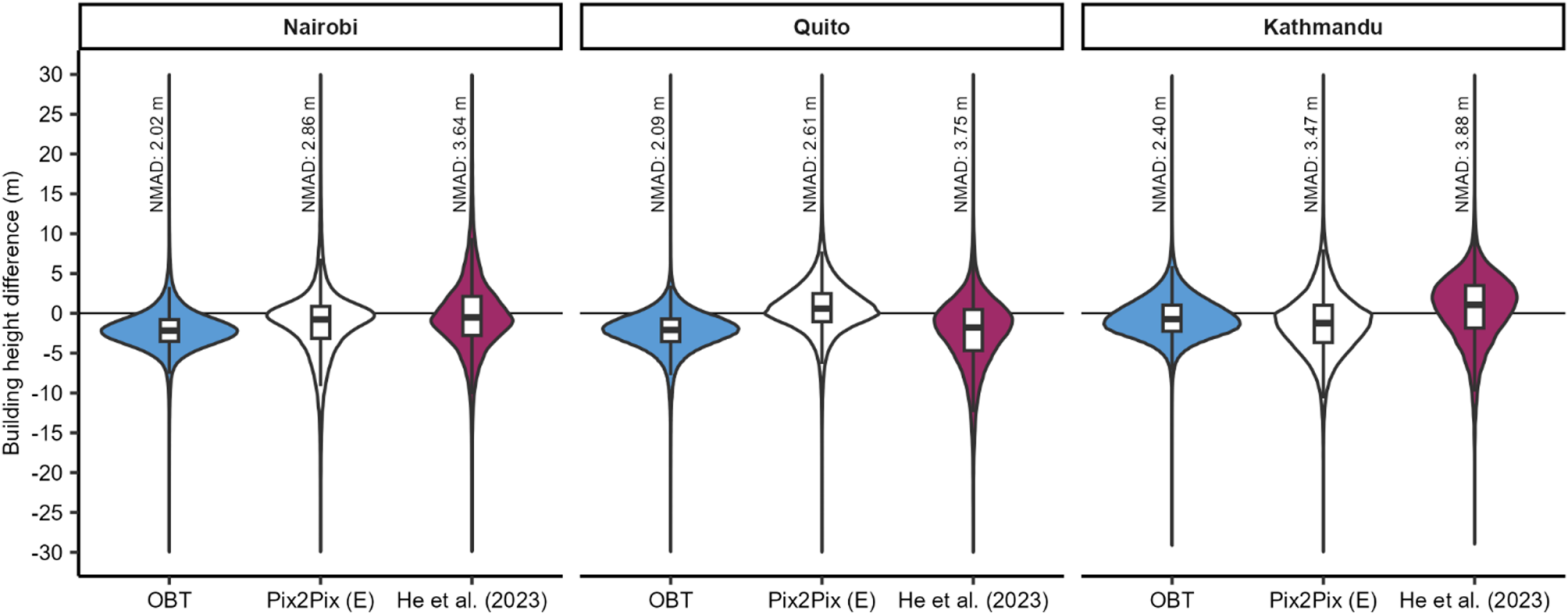
Violin and boxplots showing building height difference of the Open Buildings Temporal (blue), PixPix Model E (white), and He et al.^38^ datasets differenced from the Pleiades data.

Since the ICESat-2 reference heights were limited to 25 observations for each city, we also subtracted the gridded building height models from the Pleiades observations to derive differences. OBT predicted higher building heights compared to the Pleiades data for Nairobi and Quito (Fig. 4), with a median difference of -2.18 and − 2.09 m respectively (Table 3). This was less evident for Kathmandu, which had a median difference of -0.7 m. This trend of overprediction was similar to the comparison of building heights with the ICESat-2 data (Table 2). Overall, the error metrics including MAE and normalised median absolute deviation (NMAD) were generally lower when compared to the validation using ICESat-2 reference heights (Table 2). Errors ranged from 1.91–3.77 m MAE and 2.02–3.88 m NMAD for all models and all cities (Table 3). However, at the scale of individual buildings or clusters of buildings, notable spatial biases were apparent in the modelled building heights relative to the Pleiades satellite data (Supplementary Fig. 5). This was less apparent in the Kathmandu dataset where the distribution of building heights across the city was more homogenous with fewer tall buildings (> 50 m).

**Table 3:** Difference in modelled Building heights relative to Pleiades-derived heights.

City	Model	Median	Mean	Standard deviation	MAE	NMAD
Nairobi	Open Buildings Temporal	− 2.18	− 2.10	2.95	1.91	2.02
	Pix2Pix (E)	− 0.77	− 1.24	5.41	3.25	2.86
	He et al.^38^	− 0.49	0.07	5.92	3.77	3.64
Quito	Open Buildings Temporal	− 2.09	− 2.26	2.81	1.92	2.09
	Pix2Pix (E)	0.59	0.66	3.79	2.53	2.61
	He et al.^38^	− 1.78	− 2.28	5.21	3.58	3.75
Kathmandu	Open Buildings Temporal	− 0.73	− 0.54	2.66	2.03	2.40
	Pix2Pix (E)	− 1.23	− 1.26	4.22	3.09	3.47
	He et al.^38^	1.07	0.64	4.54	3.38	3.88

## Discussion

Our study revealed large heterogeneity in the completeness of open access building footprints for all three study cities. Building counts varied by hundreds of thousands of buildings and area coverage differed by tens of square kilometres (Fig. 1; Table 1). The disparity was also variable between the cities, with Quito featuring the largest variation across the datasets. These inconsistencies highlight the challenges in using building footprint datasets without site-specific validation. Total building counts were expected to be more variable than area coverage, since distinguishing the boundaries of individual buildings is difficult, and can be subjective when they are in close proximity, adjoining, or feature variable and connected typologies. This variation is represented in models predicting building footprints, since some will predict a single polygon for a building that is comprised of multiple connected structures, whereas others will assign a polygon for each connected element, resulting in a higher total building count and generally smaller buildings. For example, we found a smaller median building size in the GOB dataset (55–62 m^2^ compared to the GMLBF (100–530 m^2^ (Table 1). Building area coverage is more comparable and should be most affected by the acquisition date of the underlying data used to derive the inventory. The dates of this imagery are not specified for GOB and are in the range of 2014–2023 for GMLBF^31,32^. OSM is similarly biased to areas with greater availability of high-resolution satellite imagery^30,40^. In our study, satellite data was acquired within a one-year window for Quito and Kathmandu, and two years for Nairobi, which means that the building heights are temporally constrained to a known period, although new developments are still likely to have occurred in this time.

Deriving 3D city models remains an important challenge across lower- and middle-income countries, where a lack of national mapping capacity, combined with rapid urbanisation, creates dynamic cities that are not represented in open datasets^41^. This is despite the importance of building inventories across disciplines and for progressing Sustainable Development Goals^30,42^including for estimating population distributions and for disaster risk reduction^5,7,43^. High-resolution DEMs were required to derive building heights with sub-metre accuracy; however, deep learning derived models of building heights were accurate to within several metres and offer a valuable mechanism to address global inequalities in data availability^30^. However, we identified a tendency of OBT to over-predict building heights by several metres in Nairobi and Quito, highlighting the importance of site-specific validation when using large-scale global or regional datasets (Fig. 3; Table 3). Nonetheless, it was able to outperform our application of the Pix2Pix model for two of the three study cities, despite use of local satellite imagery for training. Since OBT was derived from open-access Sentinel-2 imagery, it could offer a longer-term solution to updating city models through time.

## Limitations and implications

Our comparison of open-access building footprint datasets revealed large inconsistencies in building counts and area coverage at city scales. Similarly, although we performed a local coregistration of building height datasets, differences in their creation methodologies and the variable acquisition geometry of input imagery means shifts in the apparent positions of buildings will still be present, which could bias the building height estimates for taller buildings.

The lack of ground truth building heights in our study cities, which reflect low- and middle-income countries, contributes to uncertainty. Therefore, we used both high-resolution Pleiades-derived estimates of building heights and independent ICESat-2 altimetry data for validation. However, spatial biases could still be present. For example, the Digital Terrain Model (DTM) generation procedure involves interpolating a surface between pixels identified as ground, which can be difficult to resolve in in dense urban areas, mixed with the presence of vegetation. It is also more problematic in photogrammetric DEM construction when compared to LIDAR^44,45^. Nonetheless, comparison of the city-wide DSM with ICESat-2 data demonstrated sub-metre accuracy (Supplementary Fig. 4). Additionally, we were able to identify ICESat-2 photon profiles passing over buildings and adjacent ground to derive a spatially distributed reference dataset with a mean uncertainty of 0.5 m ± 0.3 m. The global availability of ICESat-2 data means such approach is scalable and can be semi-automated^2,46^although manual inspection as used in our study may be preferred to reduce uncertainties by selecting only photons that represent a clear roof or ground return^47^.

Resolving the vertical component of cities at a large scale is becoming increasingly important and recent studies have demonstrated methodologies to achieve this at medium to coarse resolution^1,3,41^. However, closing the data gaps to provide high-resolution, building-scale height estimates for Global South countries will provide wide-ranging benefits, especially as natural hazard extremes become more prevalent. Our study demonstrates that while deep leaning methodologies can provide good predictions at city-scales, high-resolution satellite data offers the most accurate estimates. Increased acquisition and accessibility of these data over Global South cities is a priority to both inform local validation and ensure deep learning approaches do not develop and propagate biases due to the lack of Global South training data.

## Conclusions

Overall, our findings show that both building footprints and height datasets are still not well constrained spatially and temporally for our study cities in the Global South, despite the critical requirement for these data across disciplines. There is a contrast between the accessibility of these datasets in countries with established mapping agencies, and the limited accessibility of high-resolution imagery in developing countries to develop similar inventories. Our study shows the value of such data to generate DEMs with sufficient resolution to extract building heights. The Pleiades DEMs used in this study compared well to independent ICESat-2-derived building heights, which were required as validation in the absence of ground-truth data. Without aerial or LIDAR surveys, tri-stereo satellite data are an effective way of deriving city-scale high-resolution DEMs. These data also provided a unique reference dataset, allowing us to evaluate published building height datasets at a building-scale, across three cities. However, the commercial nature of this data creates access restrictions. Deep learning model predictions of building heights demonstrate that errors on the order of several metres (one building story) can be achieved at city-scales. These models can be distributed for application to new satellite imagery to update 3D city models through time without requiring new DEMs. However, spatial and temporal biases in building-scale predictions, for example related to building typology, require further investigation as 3D city models become established and used across disciplines.

### Methods

#### Study area

This study formed part of the *Tomorrow’s Cities* project, which developed a decision support framework to support pro-poor, risk-informed urban planning^37,48^. The geographic extent of the study covered the urban areas of Nairobi, Quito, and Kathmandu (Supplementary Fig. 1). The cities are exposed to a diverse range of natural hazards including earthquakes and flooding (all cities), landslides (Quito and Kathmandu), volcanic activity (Quito), and fires (Nairobi).

## DEM production and accuracy assessment

Pleiades satellites were tasked for to collect tri-stereo images over each city to produce high-resolution DEMs (Supplementary Fig. 2). Multiple acquisitions were required to capture the city extents with minimal cloud cover. Acquisitions ranged from 12/02/2020–07/03/2022 for Nairobi, 05/11/2019–28/07/2020 for Quito, and 27/10/2019–13/01/2020 for Kathmandu (Supplementary Table 3). Panchromatic (~ 0.7 m) and multi-spectral (~ 2.8 m red-green-blue and near-infrared) imagery were acquired and delivered with radiometric processing to reflectance and provided with rational polynomial coefficients (RPCs)^49,50^. Areas of cloud cover were manually masked from the analysis (Supplementary Fig. 2).

The photogrammetry software Agisoft Metashape v.2.1.1^51^ was used to generate point clouds from the tri-stereo Pleiades acquisitions. *High* quality settings, which downscales each image by a factor of four, were used to establish coincident tie points and align each image in space. First, the panchromatic and multispectral imagery were aligned in one chunk to produce a sparse point cloud. Second, the sparse cloud was then filtered to remove outliers using Metashape’s gradual selection tools to reduce the tie point root mean square error to ≤ 0.5 pixels. Third, the software generates depth maps representing the distance of each pixel from the sensor. These were used to construct a dense point cloud using the panchromatic imagery and the point cloud was used to create a 1.5 m resolution digital surface model (DSM).

The panchromatic images were pan-sharpened using the Gram-Shmidt algorithm in ArcGIS Pro v.2.8 using default settings for the Pleiades sensor and output at 0.5 m resolution. Additionally, a digital terrain model (DTM) was created from the dense point cloud using LASTools (v.13/02/2024) and the *lasground_new* tool with a 50 m step size (-metro parameter). An advantage of using tri-stereo imagery to generate elevation models in urban areas is improved ground detection amongst buildings^52^; however a large step size is required to span large buildings including warehouses for example, which were present in our study cities^53^. A trade-off is that ground detection could be overly smoothed or misdetected in on sloping ground. Therefore, the elevation difference between the DSM and DTM was used to derive the relative heights of all surface features including buildings, which we independently validated using Ice, Cloud and land Elevation Satellite (ICESat-2) laser altimetry data as described below.

ICESat-2 laser altimetry data were used to independently check the accuracy of the Pleiades-derived DEMs, since it has a higher vertical accuracy than the error expected from a Pleiades DEM created without ground control points (> 3–5 m)^54,55^. *High Confidence* returns from the Advanced Topographic Laser Altimeter System (ATLAS) instrument ATL03 Global Geolocated Photon Height data were extracted for the study areas with a date range within ± 1 year of the Pleiades acquisitions for each city^56,57^. Photons, which are transmitted and measured by the instrument approximately every 70 cm^57^were filtered to exclude slopes steeper than 20° and aggregated into mean 5 m grid cells. The Pleiades DEMs and gridded ICESAT-2 data were coregistered following the x, y, z shift correction of Nuth and Kääb^58^ and then differenced over the study areas. Forested landcover derived from ESA World Cover data^59^ was excluded from the registration and differencing since ICESat-2 would be expected to produce mixed elevation returns from both the canopy and ground, whereas Pleiades DSM would generally represent the canopy top.

### Open access Building datasets

Outlines of building footprints, which are often observed in satellite or aerial imagery as the roof footprint, are required to derive assign building-level height estimates. We compared open-access building footprint datasets for each city to identify city-scale biases in completeness, including OSM^60^Microsoft Bing’s Global ML Building Footprints (GMLBF)^32^and Google Open Buildings v3 (GOB)^31^. OSM generally contains community-contributed manually digitised building outlines^61^whereas GMLBF and GOB are derived using deep learning models applied to high-resolution satellite imagery. The precise dates of the imagery used to create the datasets are usually not reported. For example, GMLBF is extracted from imagery spanning 2014–2024, and GOB used the most recent imagery available at the time. Updates to OSM depend on the availability of data to support mapping, but also the interests of contributors^61,62^. Since the date of each dataset varies, building-level comparison are difficult and also require consideration of positional offsets between building footprints extracted from different data. Therefore, we focussed on city-scale comparison of the data. A comparison of each datasets with the temporally consistent World Settlement Footprint 2019 data is presented in Supplementary Fig. 3 to show spatial trends in completeness^63^. GOB included a relative confidence attribute and we removed buildings with a score < 0.65 to exclude the most unreliable detections^31^.

Datasets providing 3D building height information are generally aggregated to grid cells e.g. 30 m^38^ or 100 m^3,27^which can cover multiple buildings. However, the recent release of Google’s Open Buildings 2.5D Temporal dataset contains deep learning predictions of per building heights with a spatial resolution of 4 m (note that the data are upsampled to 0.5 m resolution before release^34^. We used the building presence and height data with the timestamp best aligning with the Pleiades acquisition covering the core area of each city (2021 for Nairobi, 2020 for Quito, and 2019 for Kathmandu)(Fig. 1). Additionally, we also used the global 3D urban area dataset of He et al.^38^ for comparison in our study. This dataset was gridded at 30 m resolution and was derived by combining built-up area datasets with heights assigned using a normalised ALOS World 3D DEM^38,64^. The most recent timestamp of the data was 2010, which we used in our comparison. All datasets were masked to the same built-up area extent before comparison, which is detailed in the section ‘Building height modelling and observations’.

### Building height modelling and observations

A series of Pix2Pix paired image deep learning models were trained to predict building heights from satellite imagery. The Pix2Pix approach is based on the conditional generative adversarial networks (cGAN) and uses a paired set of images, in our case a true colour satellite image and a corresponding DSM, to learn a translation between the input image and output^65^. We used Pleiades acquisitions over each city that were orthorectified using a DTM. This meant that buildings were not warped into their correct geographic position and the off nadir viewing geometry of the satellite image was preserved, which is typical of the Google Satellite Basemap imagery that is available globally. The transferability of a model trained in this way would therefore be greater, since high-resolution satellite imagery basemaps are typically not orthorectified with corresponding high-resolution DSMs. However, it means there is greater uncertainty in the alignment of building footprints between different datasets, particularly for taller buildings, which are offset depending on the off-nadir viewing geometry of the satellite.

To prepare the imagery for training the Pix2Pix models, we transformed the red-green-blue (true colour) bands of the Pleiades imagery to 8-bit unsigned integer format by clipping the minimum and maximum 0.25% of the histogram. Additionally, the DSM–DTM difference raster representing the building heights was scaled to 8-bit unsigned integer format, retaining only building heights ≤ 100 m, which was also the threshold used in the Open Buildings 2.5D Temporal dataset^34^. The Pix2Pix models were then trained on 512 × 512 pixel (256 m) chips with a 256 pixel stride (overlap), representing the true colour image and paired pixel elevations in a WGS84 coordinate system. For each model, a different combination and quantity of satellite images were used as training data from Nairobi and Kathmandu to test the impact of training data size on model quality (Supplementary Table 1). No imagery from Quito was used in the model training so this city could be used as an independent test case. The models were trained for up to 100 epochs using the ResNet-34 backbone, 10% of the training data reserved for validation, and an automatically determined optimal learning rate.

In the absence of ground-truth measurement of building heights, a reference dataset of building heights was created for 25 buildings in each city using same ICESat-2 data described earlier. We manually identified ICESat-2 profiles over buildings with both a clear ground and roof photon return. The mean elevation values of these ground and roof heights were differenced to produce a building height estimate, referred to as the ICESat-2 reference. Measurement uncertainty was derived as the square root of the sum of the squares of the standard deviation of each the roof and ground photon elevations. To maximise comparability between each dataset, we manually checked that the ICESat-2 reference points intersected the correct building in the Open Buildings 2.5D Temporal dataset. Additionally, we coregistered the Pix2Pix model inferences to using the Open Buildings 2.5D Temporal dataset using the AROSICS local image co-registration function^66^. The ICESat-2 reference building heights were compared to heights in the Open Buildings 2.5D Temporal dataset and inferences from the Pix2Pix models by sampling the mean raster elevation values in a 2 m buffer around the ICESat-2 reference measurement point. Since the ICESat-2 reference buildings represented a small sample (*n* = 25) for each city, we also compared the city-wide building height data (Fig. 2) to the Pleiades DSM-DTM heights. For the comparison with the Open Buildings 2.5D Temporal dataset, we retained *building presence* predictions with a ≥ 0.45 confidence score^34^ and used these as a mask to difference corresponding pixels in the Pleiades data. Since the dataset of He et al.^38^ was gridded at 30 m resolution, we first aggregated the same masked Pleiades data to a 30 m grid using a mean operator. Zero height values were masked from He et al.^38^ and the remaining pixels were differenced from the aggregated Pleiades data. We did not derive pixel-level comparisons with the WSF3D dataset (Fig. 2) since this dataset was gridded to 100 m and was derived by taking the median of building centroid elevations in each cell^3^meaning it was not directly comparable with the other datasets.

## Supplementary Information

Below is the link to the electronic supplementary material.

Supplementary Material 1

## Data Availability

The derived data and deep learning models will be downloadable from the Zenodo repository: [https://zenodo.org/records/13788447](https:/zenodo.org/records/13788447).
