# Supplementary material for: Narrowing the gap for city building height predictions
Source: Sci Rep. 2025 Aug 15;15:29913. doi: 10.1038/s41598-025-15929-2 (PMC12354800; doi:10.1038/s41598-025-15929-2)
Supplement: Supplementary file 1 — Supplementary Material 1 [file 41598_2025_15929_MOESM1_ESM.docx]

**Supplementary information: Narrowing the gap for city building height predictions**

C. Scott Watson^1^, John R. Elliott^2^

^1^School of Geography and water@leeds, University of Leeds, LS2 9JT, UK

^2^COMET, School of Earth and Environment, University of Leeds, LS2 9JT, UK

*Correspondence to*: C. Scott Watson (c.s.watson@leeds.ac.uk)


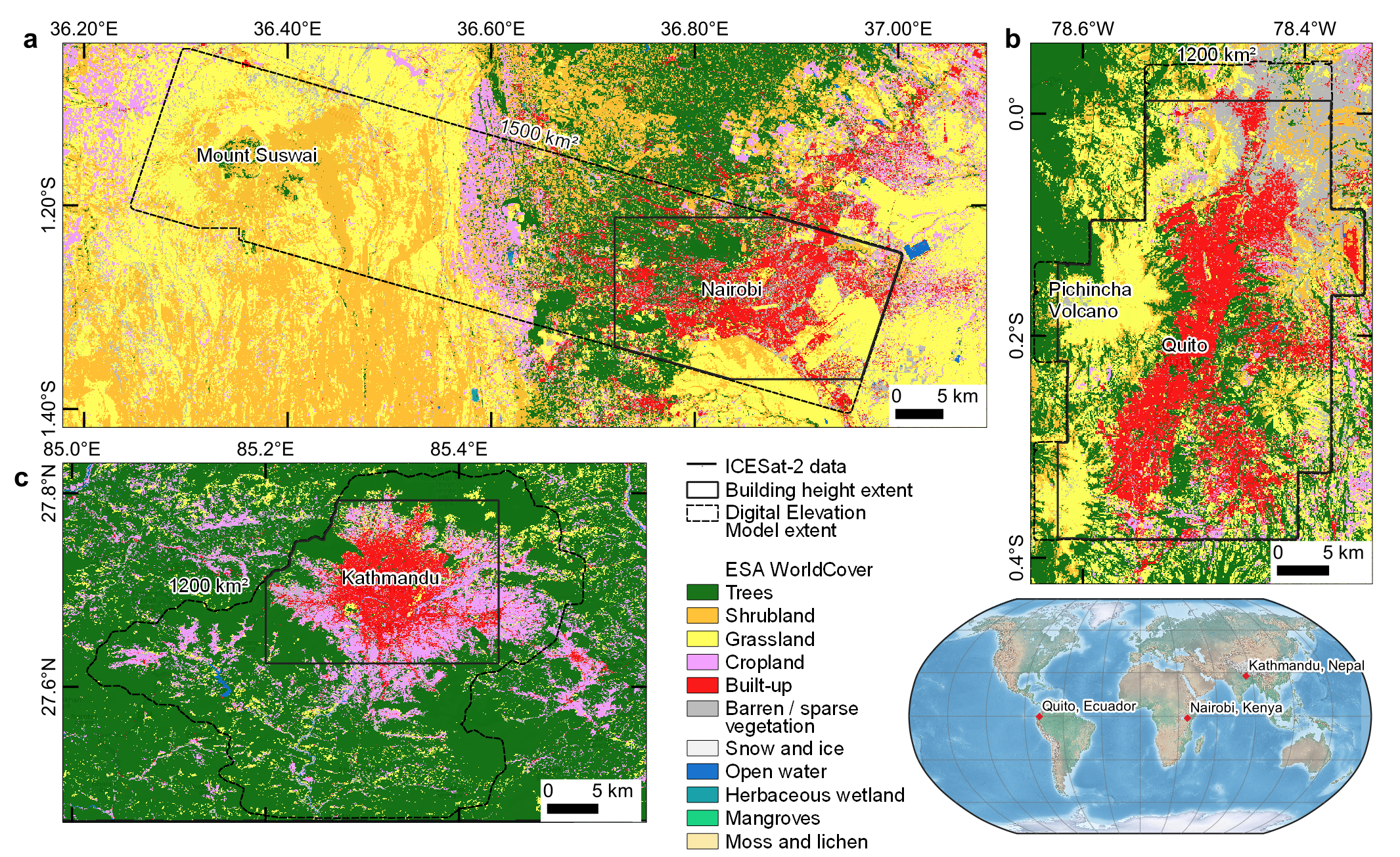


Supplementary Fig. 1 | Study areas and ESA WorldCover land cover data for each city. Panels a–c correspond to Nairobi, Kathmandu, and Quito respectively. Inset globe shows the city locations, made with Natural Earth. ESA World Cover data^1^ (© ESA WorldCover project 2020 / Contains modified Copernicus Sentinel data (2020) processed by ESA WorldCover consortium’. Figure created in QGIS 3.28.10.


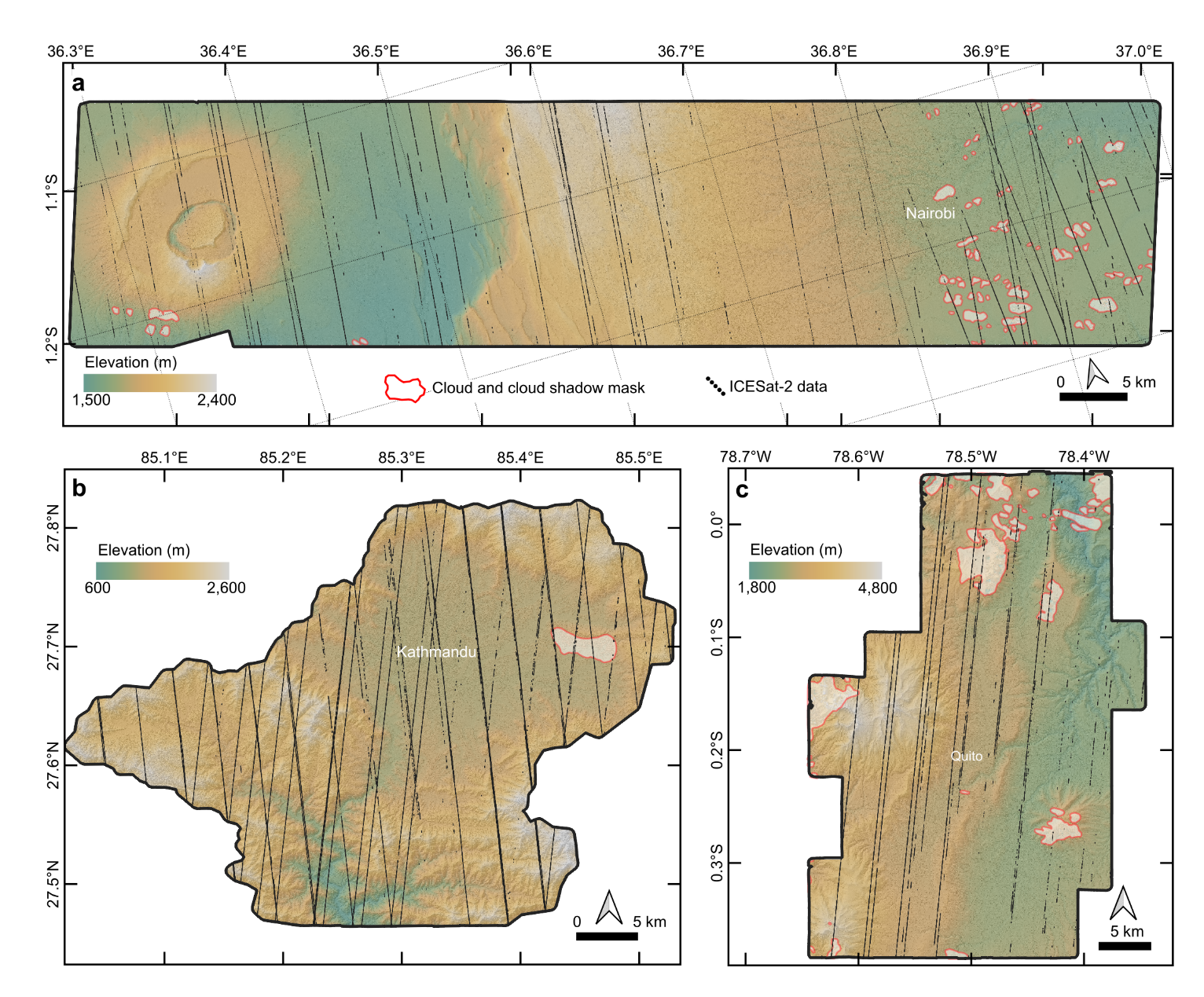


Supplementary Fig. 2 | Hillshaded digital elevation model for each city shown with the overlapping ICESat-2 data. Panels a–c correspond to Nairobi, Kathmandu, and Quito respectively. Cloud and shadow mask shows areas where elevation retrievals were limited.


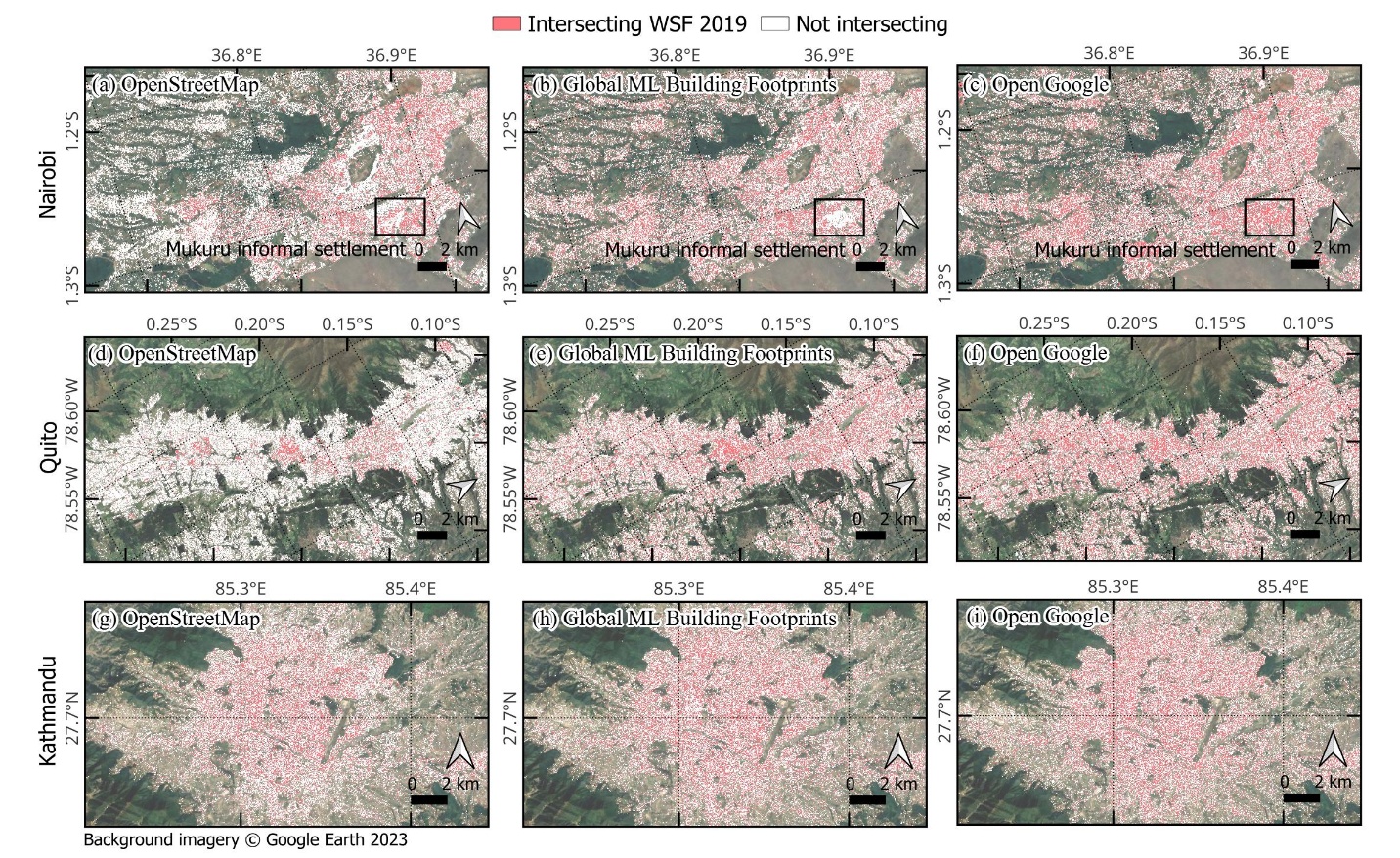


Supplementary Fig. 3 | Comparison of building footprint datasets with the World Settlement Footprint 2019 data. Intersecting areas are shown in red, and non-intersecting areas in white.

**
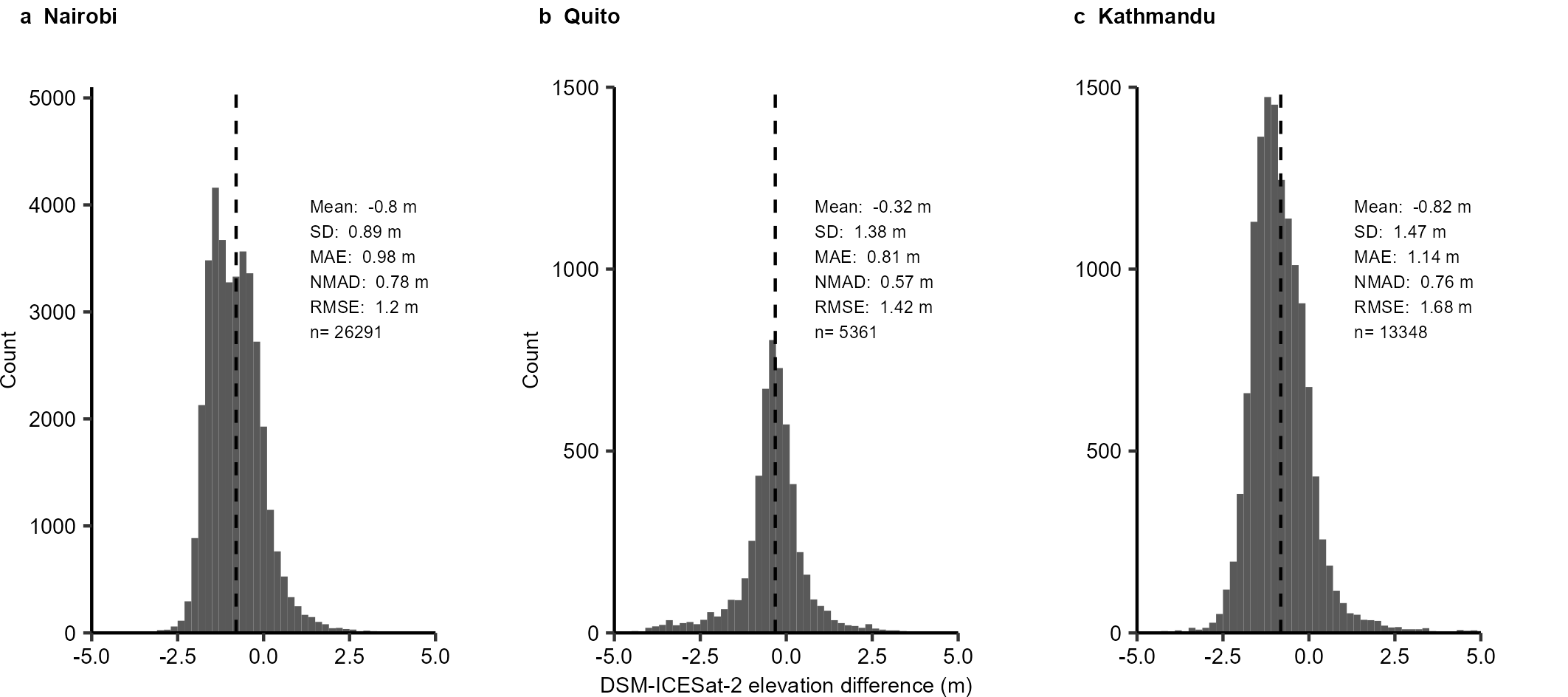
**

Supplementary Fig. 4 | Elevation differences between Pleiades DSM and ICESat-2 data.

**
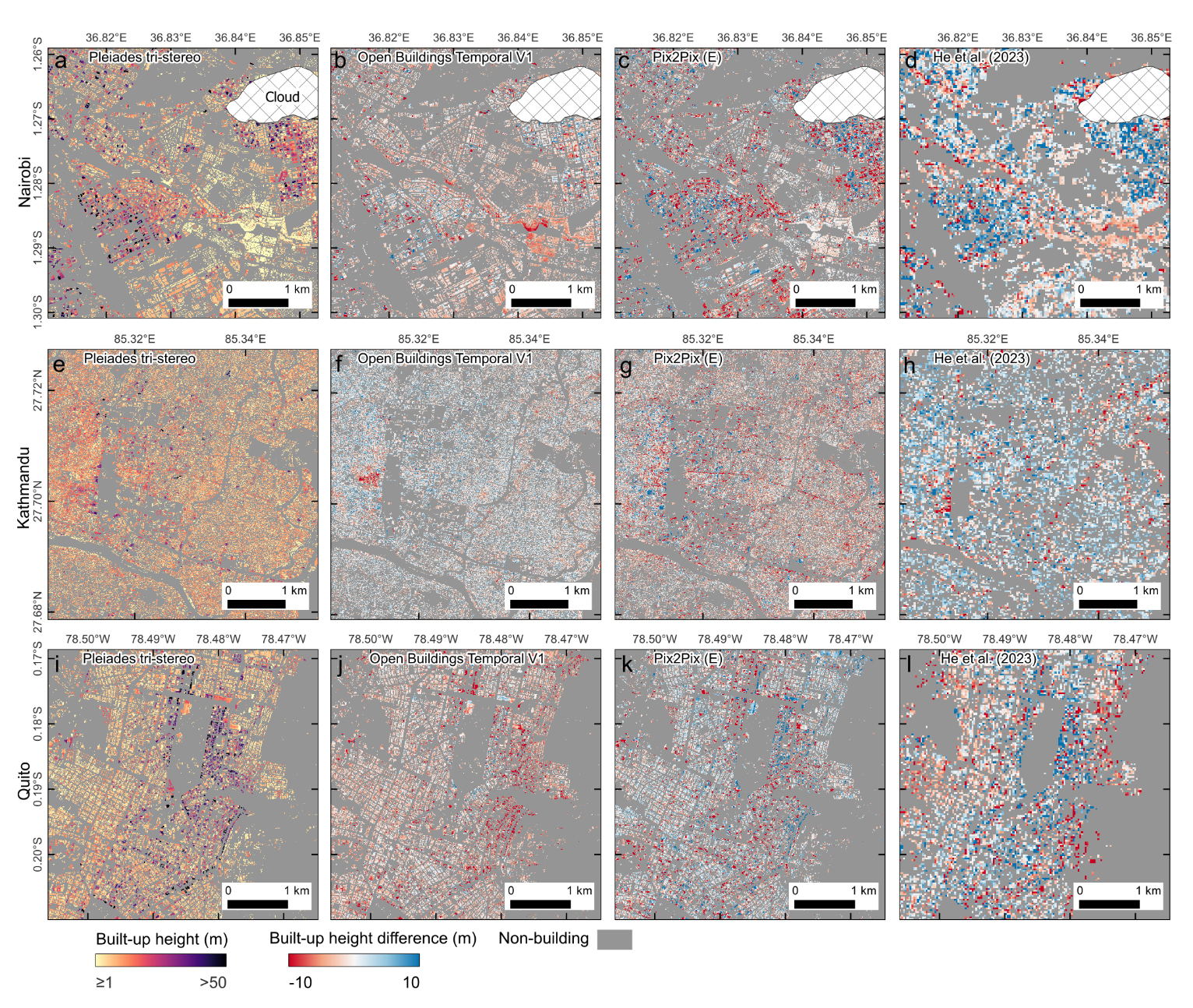
**

Supplementary Fig. 5 | Examples of built-up height difference between the datasets tested in this study and the Pleiades observations. Examples are shown for the same geographical extent as Figure 2.

Supplementary Table 1 | Imagery used to train the Pix2Pix deep learning models.

| Model ID | Training city | Number of images | Acquisition date(s) | Description |
| --- | --- | --- | --- | --- |
| A | Nairobi | 3 | 2022/Mar/07 | Model trained on one tri-stereo acquisition |
| B | Nairobi | 6 | 2022/Mar/07 and 2021/Jan/11 | Model trained on two tri-stereo acquisitions |
| C | Kathmandu | 6 | 2019/Dec/18 and 2019/Dec/25 | Model B, retrained on two tri-stereo acquisitions of Kathmandu |
| D | Kathmandu | 6 | 2019/Dec/18 and 2019/Dec/25 | Model trained on two tri-stereo acquisitions of Kathmandu |
| E | Nairobi and Kathmandu | 12 | 2022/Mar/07, 2021/Jan/11, 2019/Dec/18, and 2019/Dec/25 | Model trained on two tri-stereo acquisitions of Nairobi and Kathmandu |
| Pleiades imagery identifiers used to train the Pix2Pix models  Nairobi:  PHR1A_202203070802445_SEN_6214829101  PHR1A_ 202203070803224_SEN_6214830101  PHR1A_202203070803055_SEN_6214831101  PHR1A_202101150803351_SEN_5831364101  PHR1A_202101150802480_SEN_5831363101  PHR1A_202101150803245_SEN_5831365101  Kathmandu:  PHR1A_201912180507373_SEN_4761007101-004  PHR1A_201912180507591_SEN_4761007101-006  PHR1A_201912180508208_SEN_4761007101-005  PHR1A_201912250503529_SEN_4761008101-004  PHR1A_201912250504070_SEN_4761008101-006  PHR1A_201912250504359_SEN_4761008101-005 | | | | |

Supplementary Table 2 | Evaluation of selected building height predictions for the city of Quito with differences to the ICESat-2 reference of over 2 m.

| Building | Location (lon/lat) | Number of floors from Google Street View | ICESat-2 reference height (m) | Open Buildings Temporal (OBT) height (m) | Pleaides height (m) | Largest height difference (m) | Building description |
| --- | --- | --- | --- | --- | --- | --- | --- |
| 1 | -78.55886,  -0.30352 | 3 | 7.84 | 11.09 | 6.99 | OBT: 3.25 | Residential building block with a flat roof. Buildings of the same style on this street were estimated with heights of 11-13 m from OBT. |
| 2 | -78.55712,  -0.28637 | 2 | 6.69 | 3.13 | 6.35 | OBT:  -3.56 | Residential building with a flat roof. |
| 3 | -78.51047,  -0.22630 | Unknown | 8.73 | 15.76 | 10.50 | OBT:  7.02 | Unknown building use with flat roof. |
| 4 | -78.56080,  -0.32286 | Unknown | 5.34 | 8.01 | 4.86 | OBT: 2.67 | Residential building block adjacent to tall trees. |
| 5 | -78.55527,  -0.26799 | Unknown | 5.05 | 10.76 | 5.30 | OBT:  5.71 | Residential building block. |
| 6 | -78.47666,  -0.17508 | Unknown | 8.66 | 12.00 | 7.35 | OBT:  3.34 | Commercial building. Flat roof with raised divisions. |
| 7 | -78.47753,  -0.18377 | ~6 floors | 16.18 | 21.57 | 15.29 | OBT:  5.39 | Residential building block. |
| 8 | -78.47788,  -0.18726 | ~13 floors | 39.35 | 63.48 | 38.40 | OBT:  24.13 | High-rise residential block. Flat roof with raised section. |
| 9 | -78.49785,  -0.09992 | Unknown | 8.30 | 8.91 | 5.45 | Pleiades:  -2.85 | Residential building block |
| 10 | -78.43887,  -0.08397 | Unknown | 5.07 | 8.82 | 5.74 | OBT:  3.75 | Residential building block |
| 11 | -78.43952,  -0.09042 | 2 | 4.77 | 7.39 | 4.97 | OBT:  2.62 | Residential building block with flat roof. |
| 12 | -78.47148,  -0.12273 | 4 | 10.48 | 8.71 | 5.90 | OBT:  1.16 | Commercial/ residential block. Flat roof with raised section. |
| 13 | -78.52654,  -0.31058 | 1 | 3.49 | 6.94 | 4.01 | OBT:  1.94 | Commercial building with flat roof. |

Supplementary Table 3 | Panchromatic and multispectral Pleiades imagery identifiers that were used to generate the digital elevation models and orthoimagery.

| City | Image identifier |
| --- | --- |
| Nairobi | PHR1A_202002120802343_SEN_5831356101-1 |
|  | PHR1A_202002120802490_SEN_5831358101-1 |
|  | PHR1A_202002120803111_SEN_5831357101-1 |
|  | PHR1A_202012130806495_SEN_5831359101-1 |
|  | PHR1A_202012130807130_SEN_5831362101-1 |
|  | PHR1A_202012130807351_SEN_5831360101-1 |
|  | PHR1A_202101150802480_SEN_5831363101-1 |
|  | PHR1A_202101150803245_SEN_5831365101-1 |
|  | PHR1A_202101150803351_SEN_5831364101-1 |
|  | PHR1A_202203070802445_SEN_6214829101-1 |
|  | PHR1A_202203070802445_SEN_6214832101-1 |
|  | PHR1A_202203070803055_SEN_6214831101-1 |
|  | PHR1A_202203070803055_SEN_6214834101-1 |
|  | PHR1A_202203070803224_SEN_6214830101-1 |
|  | PHR1A_202203070803224_SEN_6214833101-1 |
|  | PHR1B_202101280803061_SEN_5831366101-1 |
|  | PHR1B_202101280803398_SEN_5831368101-1 |
|  | PHR1B_202101280803504_SEN_5831367101-1 |
| Quito | PHR1A_202001281542033_SEN_5226206101-1 |
|  | PHR1A_202001281542033_SEN_5226209101-1 |
|  | PHR1A_202001281542169_SEN_5226208101-1 |
|  | PHR1A_202001281542169_SEN_5226211101-1 |
|  | PHR1A_202001281542515_SEN_5226207101-1 |
|  | PHR1A_202001281542515_SEN_5226210101-1 |
|  | PHR1A_202002091549241_SEN_5226212101-1 |
|  | PHR1A_202002091549474_SEN_5226214101-1 |
|  | PHR1A_202002091550109_SEN_5226213101-1 |
|  | PHR1A_202006061542001_SEN_5226215101-1 |
|  | PHR1A_202006061542001_SEN_5226218101-1 |
|  | PHR1A_202006061542001_SEN_5226221101-1 |
|  | PHR1A_202006061542345_SEN_5226217101-1 |
|  | PHR1A_202006061542345_SEN_5226220101-1 |
|  | PHR1A_202006061542345_SEN_5226223101-1 |
|  | PHR1A_202006061542505_SEN_5226216101-1 |
|  | PHR1A_202006061542505_SEN_5226219101-1 |
|  | PHR1A_202006061542505_SEN_5226222101-1 |
|  | PHR1A_202007281542286_SEN_5226200101-1 |
|  | PHR1A_202007281542408_SEN_5226202101-1 |
|  | PHR1A_202007281543126_SEN_5226201101-1 |
| Kathmandu | PHR1A_201910270508003_SEN_4761010101-002 |
|  | PHR1A_201910270508103_SEN_4761010101-003 |
|  | PHR1A_201910270508408_SEN_4761010101-001 |
|  | PHR1A_201912180507373_SEN_4761007101-001 |
|  | PHR1A_201912180507591_SEN_4761007101-003 |
|  | PHR1A_201912180508208_SEN_4761007101-002 |
|  | PHR1A_201912250503529_SEN_4761008101-001 |
|  | PHR1A_201912250504070_SEN_4761008101-003 |
|  | PHR1A_201912250504359_SEN_4761008101-002 |
|  | PHR1A_202001130507364_SEN_4761009101-001 |
|  | PHR1A_202001130507474_SEN_4761009101-002 |
|  | PHR1A_202001130508143_SEN_4761009101-003 |
